# Supplementary material for: High throughput pMHC-I tetramer library production using chaperone-mediated peptide exchange
Source: Nat Commun. 2020 Apr 20;11:1909. doi: 10.1038/s41467-020-15710-1 (PMC7170893; doi:10.1038/s41467-020-15710-1)
Supplement: Supplementary file 1 — Supplementary Information [file 41467_2020_15710_MOESM1_ESM.pdf]

Supplementary Information

High Throughput pMHC-I Tetramer Library Production Using Chaperone-Mediated Peptide Exchange

Overall et al.

Supplementary Figures 1-11
Supplementary Tables 1-3
Supplementary References

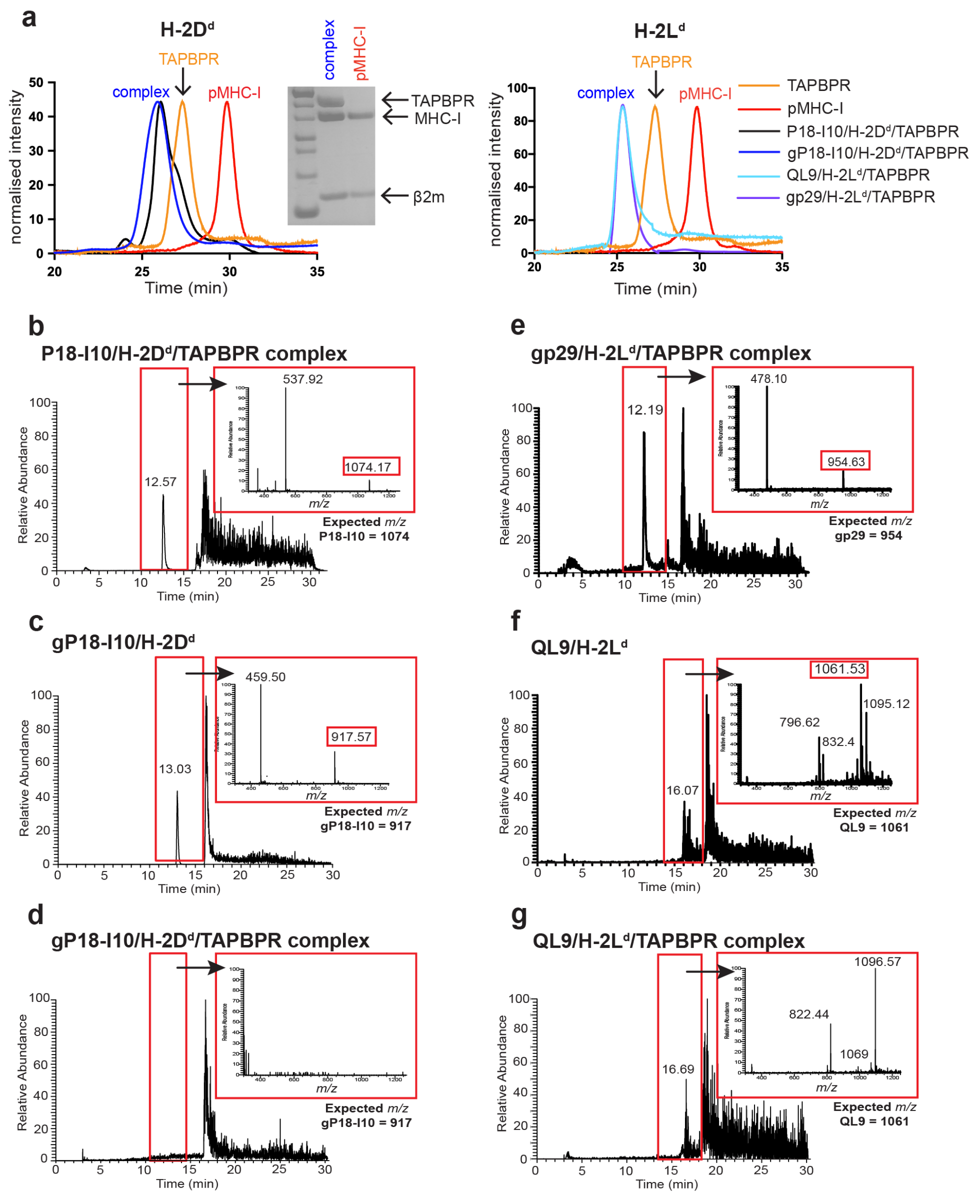

Supplementary Figure 1. Destabilizing "goldilocks" peptides promote isolation of empty murine MHC-I/TAPBPR complexes. (a) Size exclusion chromatography (SEC) elution profiles of H-2D^d bound to RGPGRAFVTI (P18-I10) or GPGRAFVTI (gP18-I10) (left panel) and H-2L^d bound to _PNVNIHNF (gp29) or QLSPFPFDL (QL9) (right panel) in the presence or absence of TAPBPR. SEC of H-2L^d molecules shown was performed in the presence of 10 mM GF dipeptide. Inset shows SDS polyacrylamide (12%) electrophoresis analysis of elution fractions at 26 min / 18 mL (pMHC-I/TAPBPR complex) and 30 min / 15 mL (pMHC-I alone). Size markers correspond to sizes of 10, 15, 20, 25, 30, 40, and 50 kDa from bottom to top, respectively. **(b-g)** Analysis of MHC-I peptide occupancy by LC-MS. Chromatograms shown are filtered to only display peaks containing *m/z* ions of interest. Insets show MS analysis of the region indicated by the red box. **(b)** P18-I10/H-2D^d/ TAPBPR complex, **(c)** gP18-I10/H-2D^d, **(d)** gP18-I10/H-2D^d/TAPBPR complex, **(e)** p29/H-2L^d/TAPBPR complex, **(f)** QL9/H-2L^d/TAPBPR complex, **(g)** QL9/H-2L^d/TAPBPR complex. Data show are representative of triplicate assays.

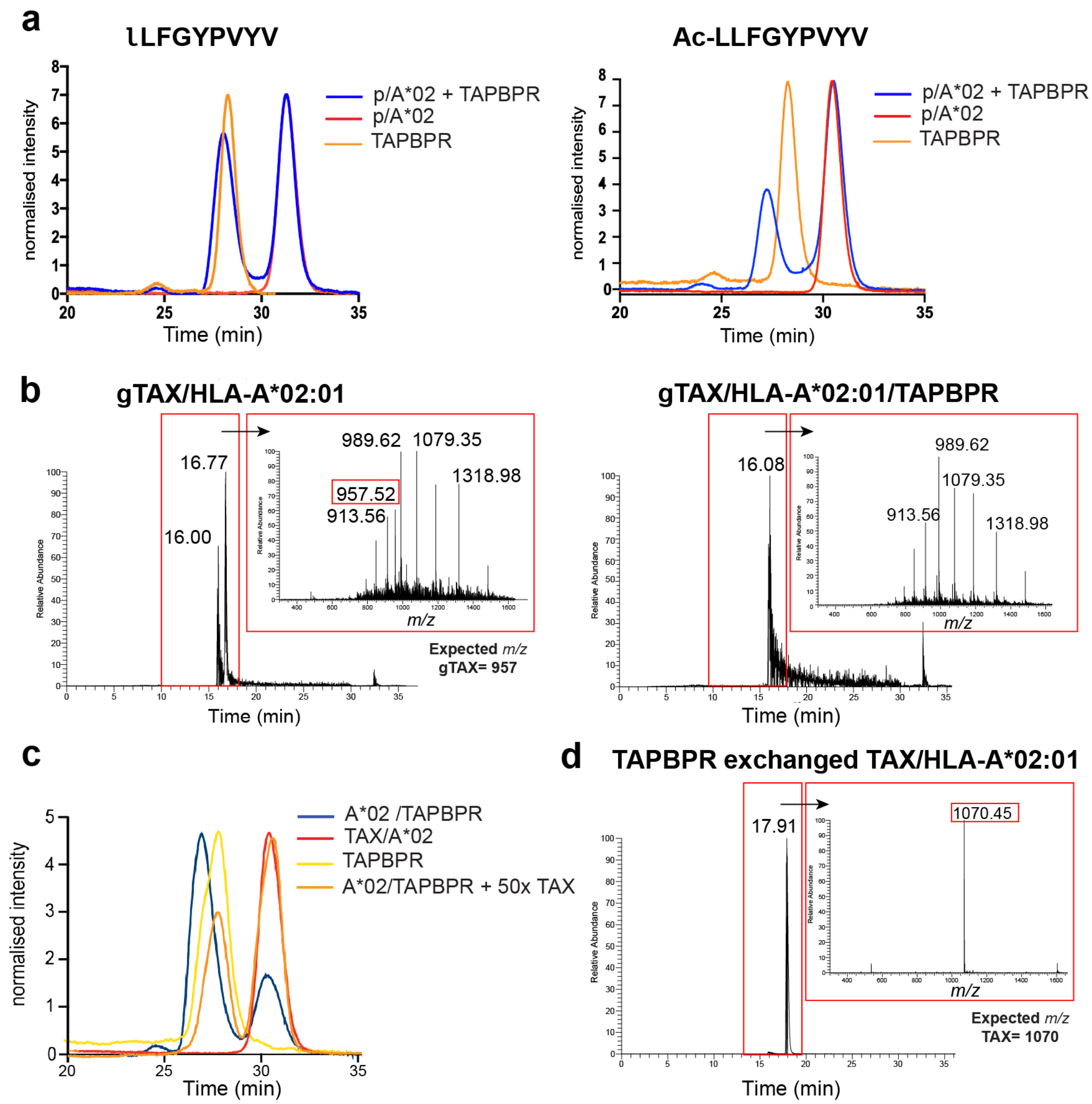

Supplementary Figure 2. Assessment of peptide occupancy of HLA-A*02:01/TAPBPR complexes. (a) SEC elution profile of lLFGYPVYV/HLA-A*02:01, where l denotes a D-Leucine residue (left), and Ac-LLFGYPVYV/HLA-A*02:01, where Ac-L denotes an acetylated N-terminal Lecuine residue (right), in the presence or absence of TAPBPR at an equimolar concentration. All binding experiments were performed in the presence of 10 mM GM dipeptide. **(b)** Analysis of peptide occupancy of gTAX/HLA-A*02:01 complex (left) and HLA-A*02:01/TAPBPR complex (right) by LC-MS. Chromatograms shown are filtered to display ions of interest. Inset: MS analysis of the region indicated. **(c)** SEC analysis of HLA-A*02:01/TAPBPR complex dissociation in the presence of high affinity (TAX) peptide. **(d)** MS analysis of TAX/HLA-A*02:01 isolated from HLA-A*02:01/TAPBPR complexes loaded with TAX. All MS analysis is done on SEC purified HLA-A*02:01. Data show are representative of triplicate assays.

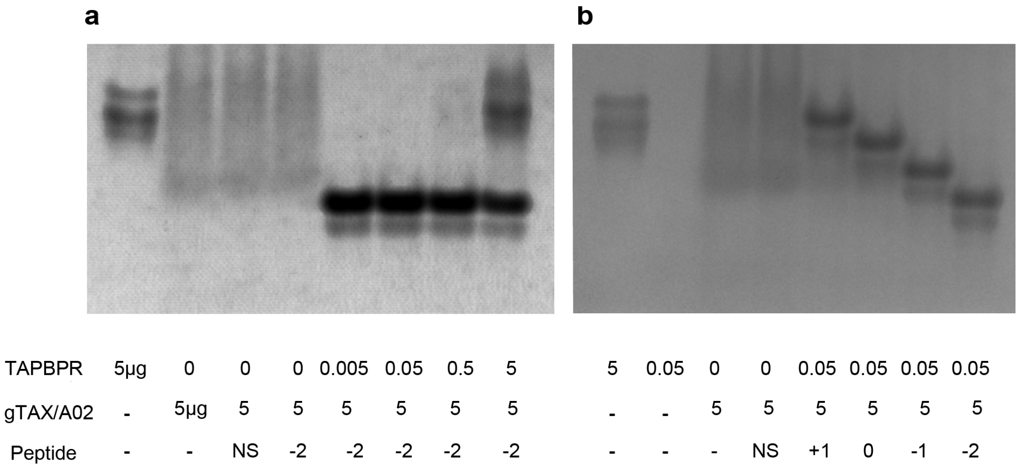

Supplementary Figure 3. Demonstration of peptide exchange using catalytic amounts of TAPBPR. (a) Native gel electrophoresis analysis of peptide exchange using varying concentrations of TAPBPR (0.005 µg to 5 µg), monitoring the formation of exchanged pMHC molecules of different electrophoretic mobilities. Each indicated reaction was incubated overnight at 4 ^{o}C in the presence of 10-fold molar excess of a high affinity peptide (sequence SLLDDAFAL, net charge of -2 at neutral pH), and a fixed concentration of gTAX/HLA-A*2:01. **(b)** Native gel electrophoresis analysis of TAPBPR exchange on gTAX/HLA-A*02:01 of four different high affinity peptides with net charges at neutral pH ranging from +1 to -2 (sequences RVADYIVKV, ALFPERITV, AIADISYSV, SLLDDAFAL), as indicated. 12% polyacrylamide native gels were run at 90V for 5 hrs at 4°C before visualization with InstantBlue (Expedeon). NS: non-specific gp29 peptide (sequence YPNVNIHNF), used as a negative control. Data shown is representative of triplicate gel assays. All protein samples used in (a) and (b) were derived from the same peptide exchange experiment, and the gels were processed in parallel.

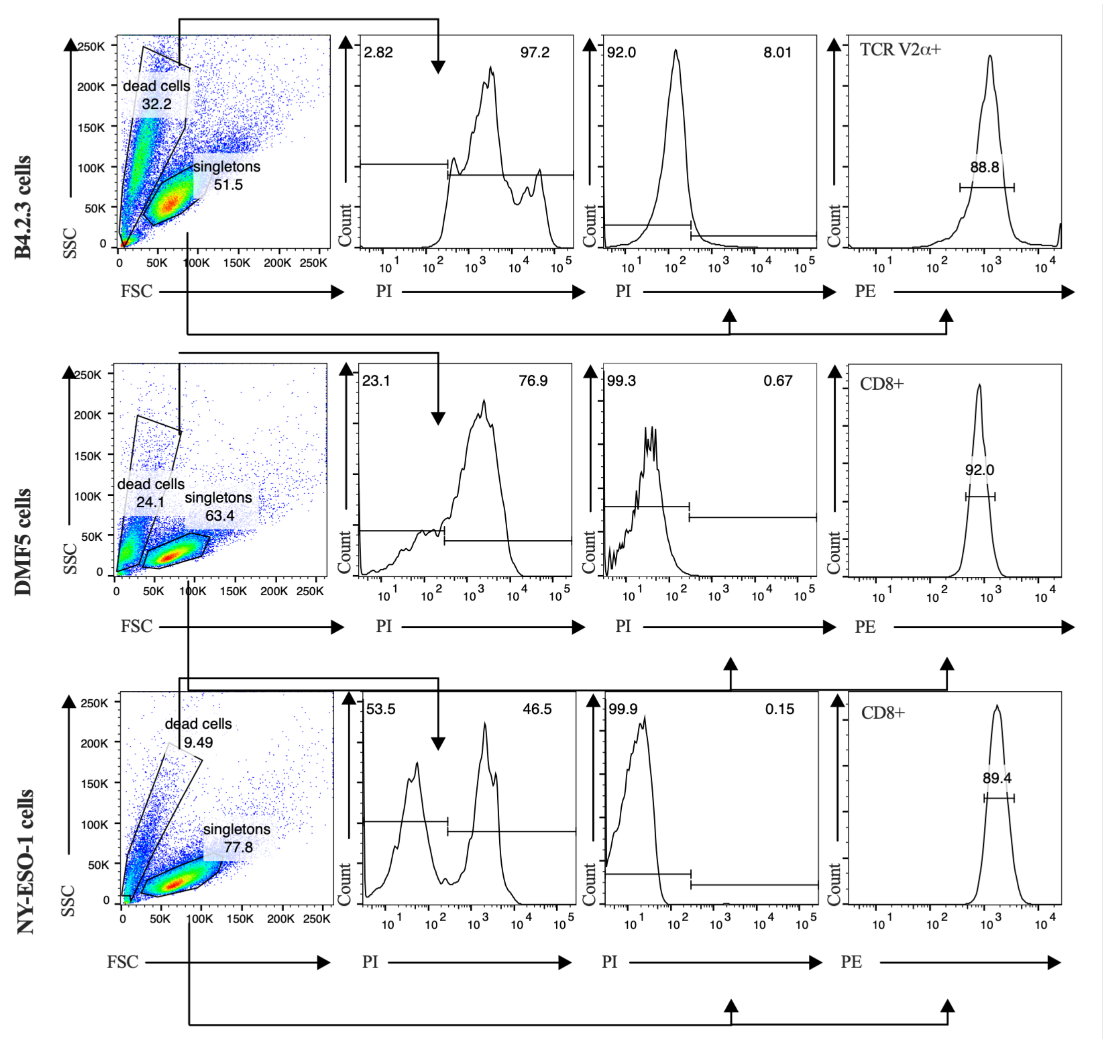

Supplementary Figure 4. Flow cytometry gating strategy for Figure 5. Acquisition and analysis was performed on an LSRII using FACSDiva software (BD, Franklin Lakes, NJ). Cells were sorted by side and forward scatter (SSC-A and FSC-A) and by propidium iodide exclusion (PE-Cy5) for viability and exclusion of multimers. Gating for single live-cells was determined by comparison of unstained and fluorescently labelled cells compensated for spectral overlap of PE, FITC and PI. Murine 58$\alpha^-\beta^-$ cells expressing the B4.2.3 TCR which recognizes P18-I10 bound to H-2D^d, were stained with BD PE-Rat Anti-Mouse Vα2 TCR Ab to confirm TCR receptor expression. The human T cell lines DMF5 and NY-ESO-1, were stained with BD FITC Mouse anti-human CD8 Ab.

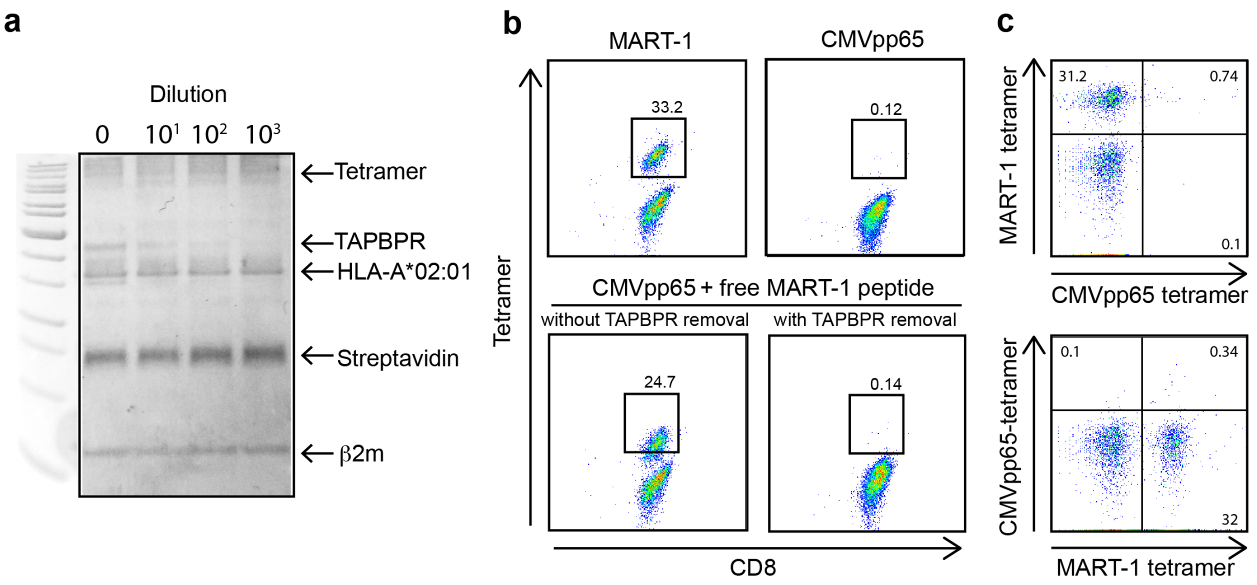

Supplementary Figure 5. Removal of TAPBPR abrogates the exchange of peptides on and between tetramers for library production. (a) SDS polyacrylamide (12%) electrophoresis of tetramers washed using a 100 kDa spin filter at 10, 100 and 1,000-fold dilutions to remove TAPBPR. Data shown is representative of triplicate gel assays. Size markers correspond to sizes of 10, 15, 20, 25, 30, 40, and 50 kDa from bottom to top, respectively. **(b)** Representative plots showing tetramer staining of DMF5 T cells with HLA-A*02:01 tetramers prepared by TAPBPR exchange of either the MART-1 epitope specific to the DMF5 TCR, or the irrelevant epitope CMVpp65 (top panels). A very low level (0.12%) of CMVpp65-tetramer positive DMF5 T cells can be detected, likely due to non-specific staining. DMF5 T cells were independently stained with CMVpp65-tetramers incubated with free MART1 peptide at 10-fold molar excess (relative to pMHC-I) without TAPBPR removal (bottom left) or upon complete removal of TAPBPR, as shown in (a) (bottom right), showing recovery of a low (0.14%) level of background staining in the absence of TAPBPR. **(c)** Staining of DMF5 T cells using a 1:1 mixture of PE-CMVpp65:APC-MART-1 tetramers (top panel), or PE-MART-1:APC-CMVpp65 tetramers (bottom panel). In each plot, both tetramer samples were prepared individually using TAPBPR exchange, followed by complete removal of TAPBPR and excess peptide, mixing of the two tetramers and overnight incubation at 4 ^{o}C. The absence of a significant PE-tetramer positive population (0.1% - x-axis) in the top panel, or an APC-tetramer positive population in the bottom pane (0.1% - y-axis) is indicative of a negligible background of cross-exchange of peptides between tetramers in the absence TAPBPR, allowing their incorporation into stable tetramer libraries. Numbers in the plots indicate the percentage of total cells. Data show are representative of triplicate, independent staining and flow cytometry experiments.

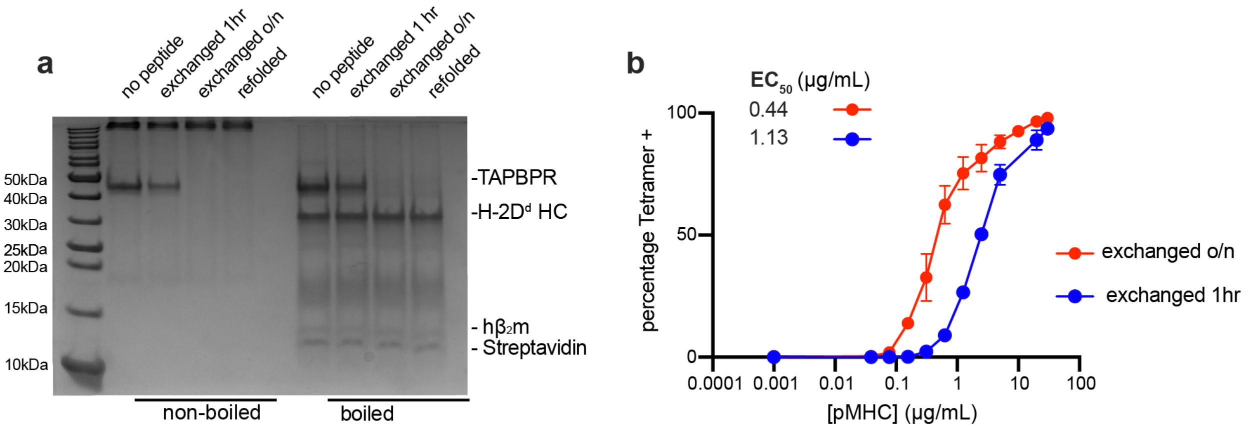

Supplementary Figure 6. Efficiency of TAPBPR-mediated loading of a high affinity peptide on H-2D^d. Tetramers produced by either 1 hr or overnight incubation of stoichiometric TAPBPR:H-2D^d complex with 10-fold molar excess of P18-I10 peptide were examined by SDS PAGE and flow cytometric staining of cells expressing B4.2.3, a TCR which recognizes P18-I10 in the context of MHC-I H-2D^d: **(a)** SDS polyacrylamide (12%) electrophoresis of tetramers assembled from pMHC-I using either protocol. Tetramers were solubilized in SDS running buffer with DTT, then boiled (as indicated) prior to electrophoresis. Refolded H-2D^d / P18-I10 and empty H-2D^d PE-tetramers were included as positive and negative controls, respectively. HC: heavy-chain. Data shown is representative of triplicate gel assays. **(b)** Titration curve of H-2D^d / P18-I10 tetramer binding to B4.2.3 cells. Tetramers were prepared using stoichiometric TAPBPR peptide loaded MHC-I following overnight (red circles) one hour (blue circles) exchange. EC$_{50}$ was calculated using a sigmoidal 4 point-plot where X equals log of concentration, using GraphPad Prism v8.0 for Mac. Error bars represent standard deviation from the mean. Data represent three independent experimental observations.

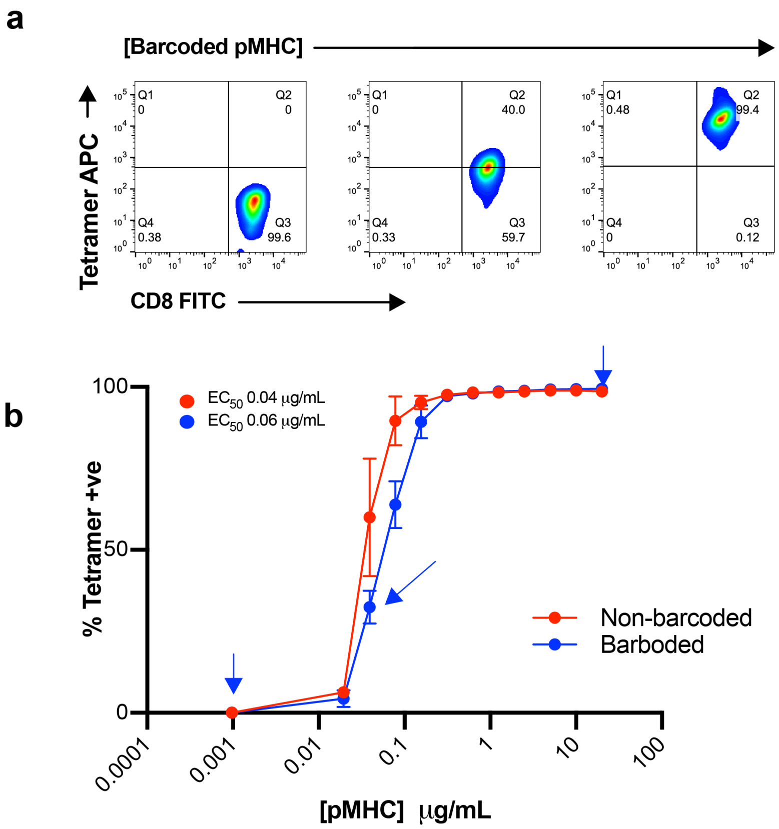

Supplementary Figure 7. Comparison of barcoded and non-barcoded tetramer binding to cognate TCR receptor. (a) Increasing amounts of MART-1-APC barcoded tetramers produced by catalytic exchange were analyzed by flow cytometric staining of cells expressing DMF5, a TCR which recognizes MART-1 in the context of HLA-A*02:01 restriction. Data shown is representative of triplicate staining assays. **(b)** Titration of bar-coded and non-barcoded tetramers. Data represents three individual flow cytometry experiments. Blue arrows indicate the concentration of pMHC-I for the three plots shown in panel (a). Percentage of cells staining positive with tetramer over a serial two-fold dilution series were plotted and EC_{50} values calculated by curve fitting to a sigmoidal line (with R2 values in the 0.97-0.99 range), using Graph Pad Prism version 8 for Mac (GraphPad Software, La Jolla California USA). Data shown is representative of triplicate assays and error-bars are standard deviation from the mean. Gating strategies used for sorting tetramer-positive cells are outlined in Supplementary Fig. 4.

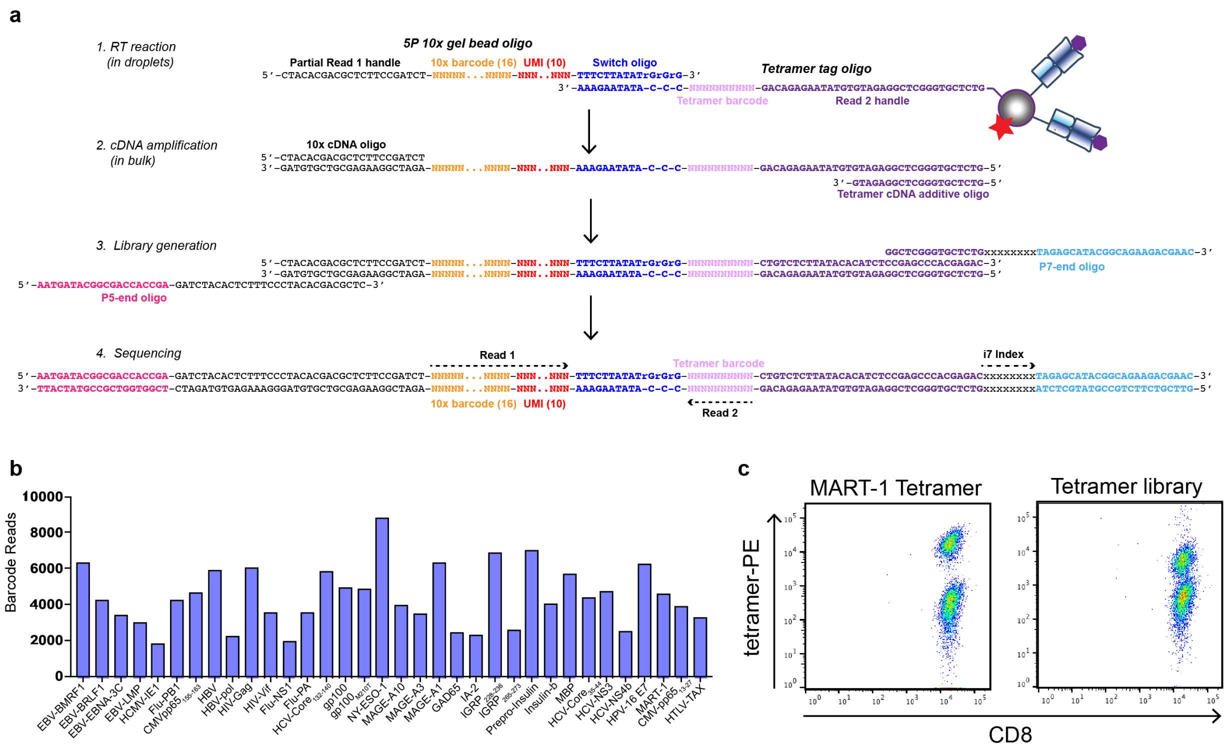

Supplementary Figure 8. ECCITE-seq adapted to capturing barcoded pMHC-I tetramers. **(a)** Biotinylated 5P ECCITE-seq oligos were conjugated to fluorophore-labelled (red star) streptavidin tetramers. The oligos contain a unique tetramer barcode, a switch oligo sequence that provides a handle for 10x compatibility by annealing of 5P 10x gel bead oligos during first-strand cDNA synthesis, in addition to a 3' Illumina NGS sequencing handle (Nextera read 2). The 10x 5P kit was used with specific protocol modifications (as outlined in Online Methods) to capture oligo-derived tags and mRNA-derived cDNA. Only oligo capture is shown here (step 1). After separation of the large and small fractions, following cDNA amplification with additive primer (step 2), the low molecular weight fraction was amplified with 10x Genomics SI-PCR oligo and a Nextera P7 oligos (step 3) to create a sequencing library compatible with Illumina instruments (step 4). The high molecular weight cDNA fraction was processed according to manufacturer's instructions. Tetramer tags and TCR cDNAs from the same cell will share the same cell barcode and can be associated. **(b)** Bulk amplification of all PE-tetramer barcodes contained in library 2. **(c)** Comparative staining of DMF5 Jurkat T cells with a single (non-barcoded) PE-tetramer prepared by TAPBPR exchange of the MART-1 peptide (left panel), versus an equal concentration of the full barcoded tetramer library 2, comprising of 34 epitopes, including MART-1 (right panel). Gating strategies are outlined in Supplementary Fig. 4. Data show are representative of triplicate, independent staining and bulk sequencing experiments.

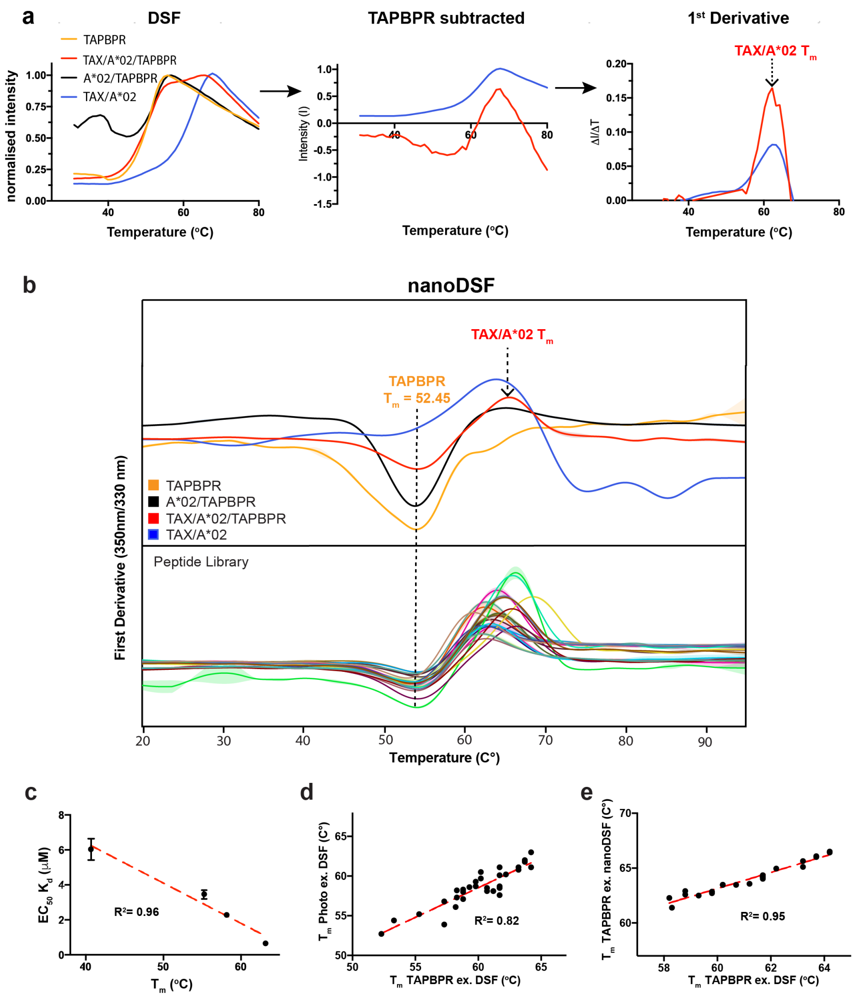

Supplementary Figure 9. High-throughput validation of peptide loading on HLA-A*02:01/TAPBPR complexes using differential scanning Fluorimetry (DSF). (a) Derivation of melting temperatures for TAPBPR exchanged TAX/HLA-A*02:01 from conventional DSF data. The DSF trace of TAPBPR alone (left, orange line) was subtracted from that of TAPBPR exchanged HLA-A*02:01 (red line) to obtain the subtracted curve (center, red line). The T_m can be extracted by taking the first derivative (right, red line). As a reference the DSF trace of TAX/HLA-A*02:01 is processed in the manner but without removing the TAPBPR trace (blue line). **(b)** (upper panel) nanoDSF traces showing a negative inflection point for TAPBPR at a T_m of 52.5 ^oC, and a positive inflection point for TAX/HLA-A*02:01 at a T_m of 66.5 ^oC. (lower panel) nanoDSF traces of HLA-A*02:01/TAPBPR loaded with different peptides from our library showing a consistent negative inflection point corresponding to the TAPBPR T_m, and a positive inflection point corresponding to the T_m values of different pMHC molecules. **(c)** Correlation between T_m values of different pHLA-A*02:01 molecules, measured by DSF, and EC_{50} values of peptide binding on empty HLA-A*02:01/TAPBPR complexes, measured by Bio-Layer Interferometry. EC_{50} error bars were estimated from 3 independent experiments per peptide. **(d)** Correlation between T_m values of pMHC molecules prepared using photo-exchange and TAPBPR-mediated peptide exchange. **(e)** Correlation between T_m values of TAPBPR exchanged pMHC molecules measured by conventional DSF as shown in (a), and by nanoDSF (b). Data shown is representative of triplicate, independent experiments.

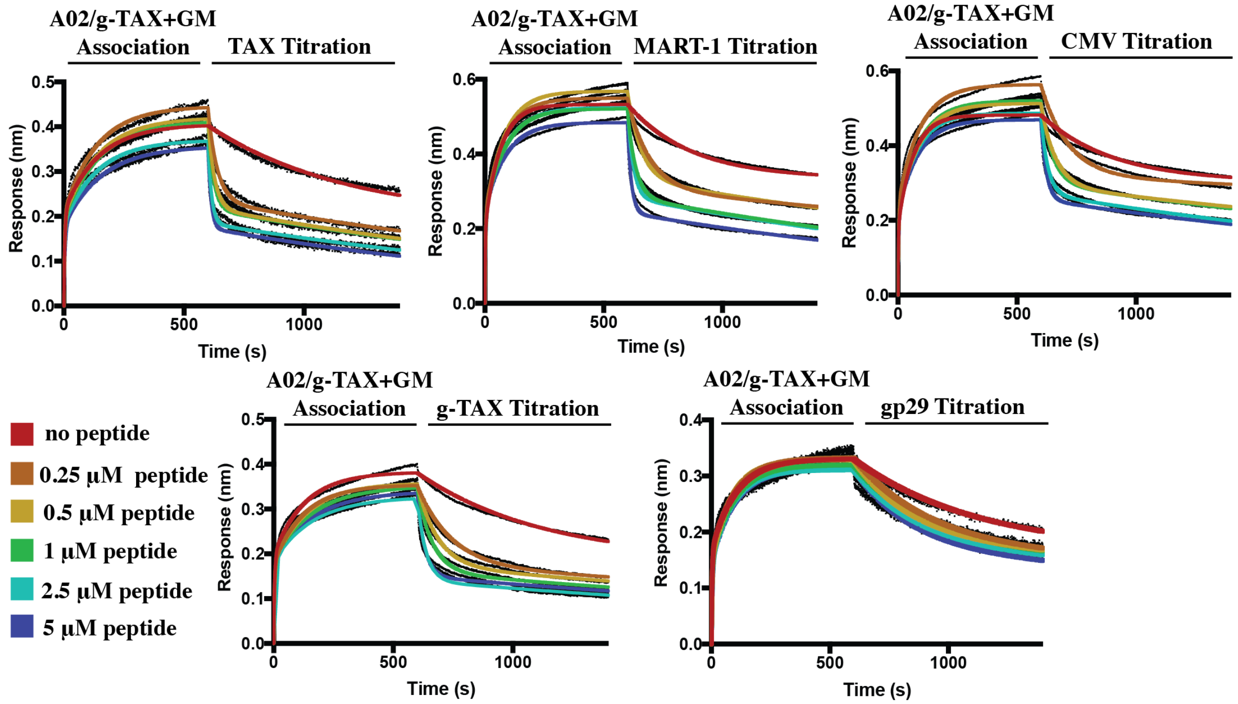

Supplementary Figure 10. Measurement of peptide-induced HLA-A*02:01 dissociation from TAPBPR using Bio-layer Interferometry. Binding and dissociation of HLA-A*02:01 from immobilized TAPBPR on a tip surface. The association of a peptide deficient HLA-A*02:01 to TAPBPR was achieved by the addition of 10 mM GM dipeptide. The dissociation of HLA-A*02:01 was measured in the presence of buffer supplemented with increasing concentrations of the indicated peptide. Dissociation data was corrected using a reference biosensor of immobilized TAPBPR (not bound to HLA-A*02:01), in buffer. Data shown are representative of 2 independent experiments. Raw data points are shown in black. All series were fit locally using a 2:1 model with a R^2 of 0.99 or greater, shown as colored curves. The derived pseudo first-order dissociation rate constants, k_d, are plotted as a function of peptide concentration in Figure 3g. Data shown is representative of triplicate, independent experiments.

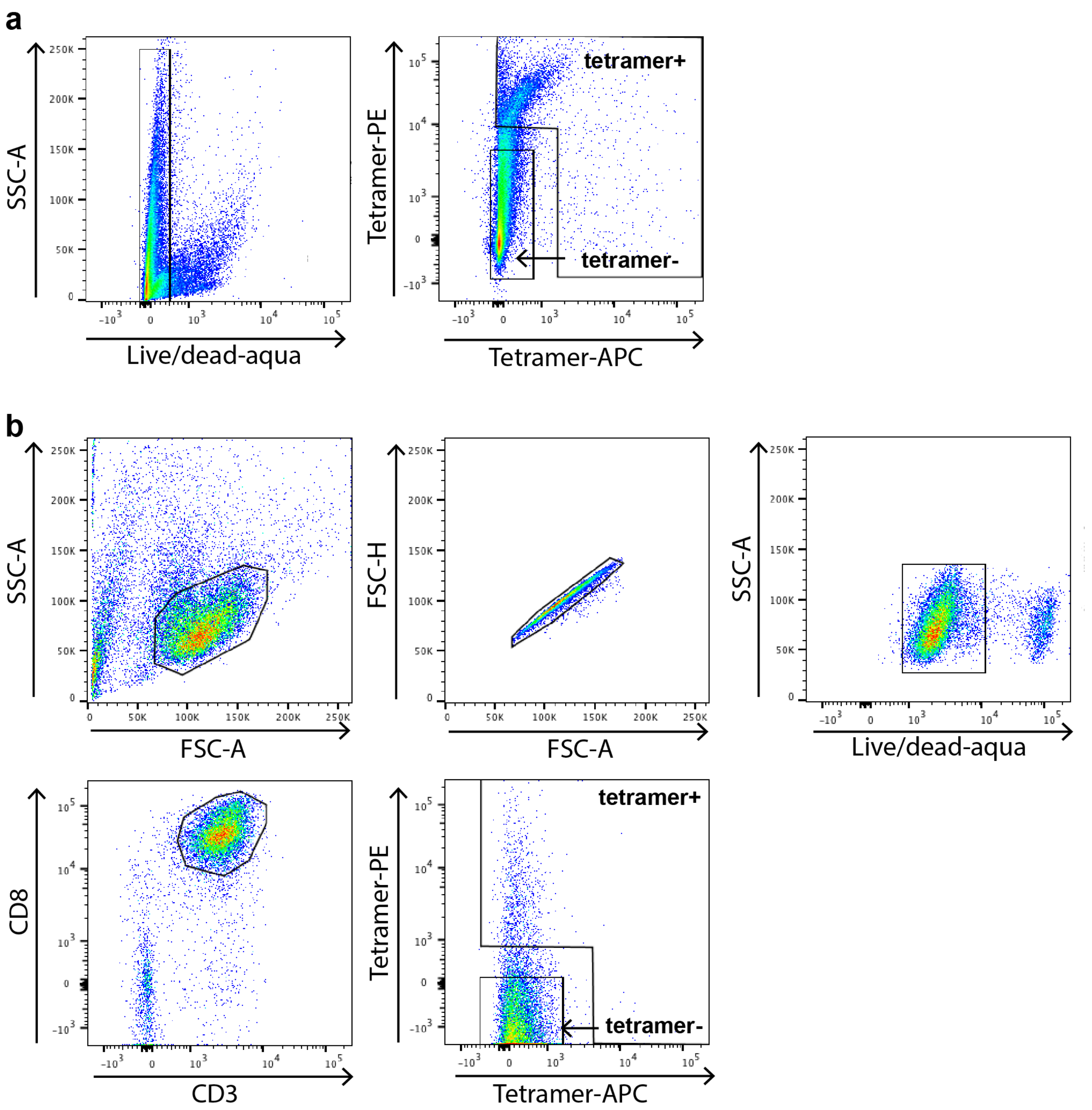

Supplementary Figure 11. Flow cytometry gating strategy. (a) Gating strategy used to sort tetramer positive cells expanded *in vitro* with NB epitope pulsed DCs. Mixed cell cultures of pulsed DCs and PBMCs were stained using both PE/APC versions of tetramer library 1, and sorted on a flow cytometer by gating on live cells, then on tetramer positive cells. **(b)** Gating strategy used to sort CD8-enriched splenocytes stained with PE/APC versions of library 2. Cells were sorted by SSC-A and FSC-A to select for lymphocytes, then filtered for single cells using FSC-H and then live cells with LIVE/DEAD-Aqua. From this, CD8+ T cells were gated based on CD8 and CD3 staining and then tetramer positive cells were collected for sequencing experiments. Due to low observed staining on the APC fluorophore channel for both samples shown in (a,b) >99% of collected cells correspond to the PE-tetramer positive fractions.

Peptide	TAPBPR exchange T_m (^oC)	photo exchange T_m (^oC)	IC_{50} (nM)
NB1	58.8 ± 0.7	57.1 ± 0.1	13
NB2	61.7 ± 0.7	58.7 ± 0.2	21
NB3	61.7 ± 0.7	57.6 ± 0.8	9
NB4	60.7 ± 0.7	58.1 ± 0.1	8
NB5	59.8 ± 0.7	59.3 ± 0.3	21
NB6	57.3 ± 0	53.9 ± 0.3	28
NB7	60.2 ± 0	59.7 ± 0.2	30
NB8	58.3 ± 0	58.2 ± 0.5	3
NB9	59.8 ± 0.7	58.7 ± 0.4	4
NB10	61.7 ± 0.7	58.5 ± 0.8	241
NB11	63.7 ± 0.7	62.0 ± 0	6
NB12	62.2 ± 1.4	60.2 ± 0.3	9
NB13	58.8 ± 0.7	58.0 ± 0.4	26
NB14	57.3 ± 1.4	56.8 ± 0.2	96
NB15	61.2 ± 0	58.1 ± 0.4	18
NB16	63.2 ± 0	61.1 ± 0.3	6
NB17	64.2 ± 0	63.0 ± 0.03	3.4
NB18	64.2 ± 0	61.1 ± 0.5	19
NB19	61.7 ± 0.7	61.1 ± 0.4	33
NB20	60.2 ± 0	60.5 ± 0.1	42
NB21	58.8 ± 0.7	58.3 ± 0.2	48
NB22	59.3 ± 0	58.6 ± 0.1	13
NB23	60.7 ± 0.7	58.5 ± 0.5	13
NB24	63.2 ± 1.4	60.8 ± 0.3	3
NB25	63.7 ± 0.7	61.8± 0.3	14
NB26	61.7 ± 0.7	60.1± 0.1	19
NB27	53.3 ± 0	54.4± 0.2	480
NB28	52.3 ± 0	52.7± 0.2	635
NB29	58.3 ± 0	57.3± 0.1	4
MART1 (reference)	58.2 ± 0	56.1± 0.5	254

Supplementary Table 1. pMHC T_m values of Neuroblastoma neoepitopes included in Library 1. Conventional DSF was performed on pHLA- A*02:01 samples prepared by either TAPBPR-mediated peptide exchange (column 2), or exchange using UV irradiation of a photo-sensitive conditional peptide ligand (column 3), as outlined in Online Methods. A 20-fold molar excess of free peptide was used to promote exchange during a 1 hr incubation at room temperature, for all experiments. DSF profiles were analysed as shown in Supplementary Fig. 9a. All measurements were performed in PBS buffer. Errors represent the standard deviation of 3 replicates, individually analysed. IC_{50} values were obtained from NetMHCpan-4.0 [6].

Peptide	Epitope	Sequence	Tm (°C)	IC_{50}
EBV BMRF1	208-216	TLDYKPLSV	51.3 ± 0	36
EBV BRLF1	109-117	YVLDHLIVV	55.3 ± 0	4
EBV EBNA3c	284-293	LLDFVRFMGV	55.3 ± 3.0	54
EBV LMP-1	159-167	YLQQNWWTL	54.3 ± 0	9
HCMV IE1	81-89	VLAELVKQI	50.3 ± 1.0	154
Influenza PB1	413-421	NMLSTVLGV	60.2 ± 0	10
HCMV pp65	155-163	QMWQARLTV	53.3 ± 0	81
HBV core	19-27	FLFPSDFFPSV	62.2 ± 0	4
HBV Pol	575-583	FLLSLGIHL	53.3 ± 1.0	9
HIV Gag	77-85	SLYNTVATL	50.8 ± 0.5	54
HIV Vif	101-109	GLADQLIHL	60.2 ± 0	10
Influenza NS1	1122-1130	AIMDKNIIL	52.8 ± 0.5	54
Influenza PA	70-78	ALLKHRFEI	53.3 ± 0	37
HCV Core	132-140	DLMGYIPAV	52.3 ± 0	10
gp100	209-217	IMDQVPFSV	54.3 ± 0	6
gp100	209-217 (210T)	ITDQVPFSV	50.3 ± 0	102
NY-ESO-1	157-165	SLLMWITQA	51.3 ± 0	26
MAGE-A10	254-262	GLYDGMEHL	53.3 ± 0	9
MAGE-A3	271-279	FLWGPRALV	53.3 ± 0	12
MAGE-A1	278-286	KVLEYVIKV	61.7 ± 0.5	7
GAD65	114-122	VMNILLQYV	47.3 ± 0	23
IA-2	805-813	VIVMLTPLV	47.8 ± 0.5	72
IGRP	228-236	LNIDLLWSV	53.8 ± 0.5	243
IGRP	265-273	VLFGLGFAI	50.3 ± 0	16
Prepro-insulin	15-24	ALWGPDPAAA	53.3 ± 0	234
Insulin b	10-18	HLVEALYLV	61.2 ± 0	7
MBP	110-118	SLSRFSWGA	47.8 ± 0.5	24
HCV core	35-44	YLLPRRGPRL	53.3 ± 0	245
HCV NS3	1406-1415	KLSGLGINAV	60.2 ± 0	41
HCV NS4b	1807-1816	LLFNILGGWV	55.3 ± 0	49
HPV16 E7	12-20	MLDLQPETT	43.9 ± 0.5	1812
CMV pp65	496-503	NLVPMVATV	55.3 ± 0	25
HTLV	11-19	LLFGYPVYV	61.2 ± 0	3
MART1 (reference)	26-35	ELAGIGILTV	58.2 ± 0	254

Supplementary Table 2. pMHC T_m values of viral, tumor, autoimmune epitopes included in Library 2. Conventional DSF was performed on HLA-A*02:01/TAPBPR complexes following 1 hr incubation with each listed peptide. A 20-fold molar excess of free peptide was used to promote

exchange during a 1 hr incubation at room temperature, for all experiments. DSF profiles were analysed as shown in Supplementary Fig. 9a. Measurements were performed in PBS buffer supplemented with 0.5% DMSO to increase peptide solubility. Errors represent the standard deviation of 3 replicates, individually analysed. IC_{50} values were obtained from NetMHCpan-4.0[6].

Oligo	Sequence
Tetramer barcode oligos	/5biosg/GTCTCGTGGGCTCGGAGATGTGTATAAGAGACAG xxxxxxxxxxxxCCCATATAAGAAA
tetramer additive	GTCTCGTGGGCTCGGAGATG
DMF5_PCR1	GAAATTCACGGCGCACAGG
DMF5_PCR2	CCTTGGCACCCGAGAATTCCAGCTTGGCTGGCTGTCTCTGATC
P5 Generic	AATGATACGGCGACCACCGAGATCTACAC
RPIx	CAAGCAGAAGACGGCATACGAGATxxxxxxxxGTGACTGGAGTT CCTTGGCACCCGAGAATTCCA
Tet_bulk_oligo	CTACACGACGCTCTTCCGATCTNNNNNNNNNNTTTCTTATATGGG
10x_5P_RT	AAGCAGTGGTATCAACGCAGAGTACGAGACTTTTTTTTTTTTTTT TTTTTTTTTTTTTTVN
N7xx	CAAGCAGAAGACGGCATACGAGATxxxxxxxxGTCTCGTGGGCTCGG
10x_PCR_F	CTACACGACGCTCTTCCGATCT
10x_PCR_R	AAGCAGTGGTATCAACGCAGAGTACAT
10x_SI_PCR	AATGATACGGCGACCACCGAGATCTACACTCTTTCCCTACACGACGC*T*C
10x Chromium i7 index	CAAGCAGAAGACGGCATACGAGATxxxxxxxxGTGACTGGAGTTCAGACGTGT
10x Human T Cell Mix 1 F	AATGATACGGCGACCACCGAGATCTACACTCTTTCCCTACACGACGCTC
10x Human T Cell Mix 1 R	TGAAGGCGTTTGCACATGCA
10x Human T Cell Mix 1 R	TCAGGCAGTATCTGGAGTCATTGAG
10x Human T Cell Mix 2 F	AATGATACGGCGACCACCGAGATCT
10x Human T Cell Mix 2 R	AGTCTCTCAGCTGGTACACG
10x Human T Cell Mix 2 R	TCTGATGGCTCAAACACAGC

Supplementary Table 3. Primer sequences. Primer sequences used for ECCITE-seq analysis.

Supplementary References

1. Natarajan, K. *et al.* An allosteric site in the T cell receptor Cβ domain plays a critical signalling role. *Nature Communications* **8**, 15260 (2017).
2. Balendiran, G. K. *et al.* The three-dimensional structure of an H-2Ld-peptide complex explains the unique interaction of Ld with beta-2 microglobulin and peptide. *Proc Natl Acad Sci U S A* **94**, 6880–6885 (1997).
3. Adams, J. J. *et al.* T cell receptor signaling is limited by docking geometry to peptide-major histocompatibility complex. *Immunity* **35**, 681–693 (2011).
4. Khan, A. R., Baker, B. M., Ghosh, P., Biddison, W. E. & Wiley, D. C. The structure and stability of an HLA-A*0201/octameric tax peptide complex with an empty conserved peptide-N-terminal binding site. *J. Immunol.* **164**, 6398–6405 (2000).
5. Jiang, J. et al. Crystal structure of a TAPBPR–MHC I complex reveals the mechanism of peptide editing in antigen presentation. Science 358, 1064-1068 (2017).
6. Jurtz, V. *et al*. NetMHCpan-4.0: Improved peptide–MHC class I interaction predictions integrating eluted ligand and peptide binding affinity data. *The Journal of Immunology* **199**, 3360-3368 (2017).
