# Supplementary material for: High throughput pMHC-I tetramer library production using chaperone-mediated peptide exchange
Source: Nat Commun. 2020 Apr 20;11:1909. doi: 10.1038/s41467-020-15710-1 (PMC7170893; doi:10.1038/s41467-020-15710-1)
Supplement: Supplementary file 3 — Reporting Summary [file 41467_2020_15710_MOESM3_ESM.pdf]

nature research

Corresponding author(s): Nikolaos G. Sgourakis, Ph.D.

Last updated by author(s): 3/12/2020

Reporting Summary

Nature Research wishes to improve the reproducibility of the work that we publish. This form provides structure for consistency and transparency in reporting. For further information on Nature Research policies, see Authors & Referees and the Editorial Policy Checklist.

Statistics

For all statistical analyses, confirm that the following items are present in the figure legend, table legend, main text, or Methods section.

n/a	Confirmed	
☐	☒	The exact sample size (*n*) for each experimental group/condition, given as a discrete number and unit of measurement
☐	☒	A statement on whether measurements were taken from distinct samples or whether the same sample was measured repeatedly
☐	☒	The statistical test(s) used AND whether they are one- or two-sided<br>*Only common tests should be described solely by name; describe more complex techniques in the Methods section.*
☒	☐	A description of all covariates tested
☒	☐	A description of any assumptions or corrections, such as tests of normality and adjustment for multiple comparisons
☒	☐	A full description of the statistical parameters including central tendency (e.g. means) or other basic estimates (e.g. regression coefficient) AND variation (e.g. standard deviation) or associated estimates of uncertainty (e.g. confidence intervals)
☐	☒	For null hypothesis testing, the test statistic (e.g. F, t, r) with confidence intervals, effect sizes, degrees of freedom and P value noted<br>*Give P values as exact values whenever suitable.*
☒	☐	For Bayesian analysis, information on the choice of priors and Markov chain Monte Carlo settings
☒	☐	For hierarchical and complex designs, identification of the appropriate level for tests and full reporting of outcomes
☐	☒	Estimates of effect sizes (e.g. Cohen's *d*, Pearson's *r*), indicating how they were calculated

Our web collection on statistics for biologists contains articles on many of the points above.

Software and code

Policy information about availability of computer code

Data collection	For the single cell gene expression and clonotype data, raw reads were processed into counts matrices using 10x cell ranger pipelines: https://support.10xgenomics.com/single-cell-gene-expression/software/pipelines/latest/what-is-cell-ranger<br>Tag counts for MHC multimers were processed with CITE-seq count: https://github.com/Hoohm/CITE-seq-Count<br>For Flow Cytometry data collection, we used BD FACSDiva™ Software v6, Becton Dickinson, San Jose Ca. For Bio-layer interferometry we used the Octet data analysis HT v.11.1 software. For collection of DSF data, we used the PR Control software v1 (NanoTemper Technologies).
Data analysis	To identify the DMF5 tetramer, we used CITE-seq-Count version 1.4.1 to search for the DMF5 specific tag, using default parameters (hamming distance set to 5). For the tetramer library, we used CITE-seq-Count version 1.4.1 using all default parameters, with the exception of hamming distance set to 1, and a cell-barcode whitelist to limit the search to cells with T cell receptor sequences found by the 10X pipeline. For flow cytometry Data analysis, we used FlowJo v10, BD Biosciences, CA. For Bio-layer interferometry data analysis we used the Octet data analysis HT v.11.1 software. For analysis of nanoDSF data, we used the PR Control software v1.0 (NanoTemper Technologies). For supplementary analysis of all data we used GraphPad Prism v8.0 for Mac, GraphPad Software, Inc.

For manuscripts utilizing custom algorithms or software that are central to the research but not yet described in published literature, software must be made available to editors/reviewers. We strongly encourage code deposition in a community repository (e.g. GitHub). See the Nature Research guidelines for submitting code & software for further information.

Data

Policy information about availability of data

All manuscripts must include a data availability statement. This statement should provide the following information, where applicable:

- Accession codes, unique identifiers, or web links for publicly available datasets
- A list of figures that have associated raw data
- A description of any restrictions on data availability

Raw and processed Illumina sequencing data are deposited with National Center for Biotechnology Information’s Gene Expression Omnibus under accession

codes GSE146826, GSM4407564, GSM4407565, GSM4407566 and GSM4407567.
The datasets generated and analysed during the current study are available from the corresponding author on reasonable request.

Field-specific reporting

Please select the one below that is the best fit for your research. If you are not sure, read the appropriate sections before making your selection.

☒ Life sciences ☐ Behavioural & social sciences ☐ Ecological, evolutionary & environmental sciences

For a reference copy of the document with all sections, see nature.com/documents/nr-reporting-summary-flat.pdf

Life sciences study design

All studies must disclose on these points even when the disclosure is negative.

Sample size

For all datasets provided in this work, we chose replicate sizes according to standard methods provided in the literature:
Biochemical peptide exchange assays:

Morozov, G. I. et al. Interaction of TAPBPR, a tapasin homolog, with MHC-I molecules promotes peptide editing. Proceedings of the National Academy of Sciences 113, E1006-E1015 (2016).

McShan, A. C. et al. Peptide exchange on MHC-I by TAPBPR is driven by a negative allostery release cycle. Nature chemical biology 14, 811 (2018).

McShan, A. C. et al. Molecular determinants of chaperone interactions on MHC-I for folding and antigen repertoire selection. Proc Natl Acad Sci U S A 116, 25602-25613, doi:10.1073/pnas.1915562116 (2019).

Tetramer binding assays:
Altman, J. D. et al. Phenotypic analysis of antigen-specific T lymphocytes. Science 274, 94-96 (1996).

Bentzen, A. K. et al. Large-scale detection of antigen-specific T cells using peptide-MHC-I multimers labeled with DNA barcodes. Nature biotechnology 34, 1037 (2016).

Single-cell analysis:
Mimitou, E. P. et al. Multiplexed detection of proteins, transcriptomes, clonotypes and CRISPR perturbations in single cells. Nat Methods 16, 409-412, doi:10.1038/s41592-019-0392-0 (2019).

All details are thoroughly cited in methods and in figure legends.

Data exclusions

No data were excluded from the analyses.

Replication

Independent, triplicate experiments using freshly prepared reagents were used in all biochemical assays provided. General measures taken to verify the reproducibility of experiments included independent staining experiments by different researchers in 3 different laboratories within our team.

Randomization

All data reported here were recorded using purified samples of recombinantly expressed molecules in buffered solutions, in contrast to experiments using animal models. As a direct consequence of our approach, there is no need for randomization of samples, and as such it was not pursued in this study.

Blinding

All data reported here were recorded using purified samples of recombinantly expressed molecules in buffered solutions, in contrast to experiments using animal models. As a direct consequence of our approach, blinding was not relevant to this study.

Reporting for specific materials, systems and methods

We require information from authors about some types of materials, experimental systems and methods used in many studies. Here, indicate whether each material, system or method listed is relevant to your study. If you are not sure if a list item applies to your research, read the appropriate section before selecting a response.

Materials & experimental systems

n/a	Involved in the study	
☐	☒	Antibodies
☐	☒	Eukaryotic cell lines
☒	☐	Palaeontology
☒	☐	Animals and other organisms
☒	☐	Human research participants
☒	☐	Clinical data

Methods

n/a	Involved in the study	
☒	☐	ChIP-seq
☐	☒	Flow cytometry
☒	☐	MRI-based neuroimaging

Antibodies

Antibodies used

FITC Mouse anti-CD8 clone G42-8, BD, San Jose CA PE Rat anti-Mouse TCR Va2 clone B20.1 BD, San Jose CA

Validation

Clone G42.8 validated for FACS on manufacturers website as staining 13-48% of peripheral blood lymphocytes and 80% of thymocytes and a subset of NK.

Schlossman SF. Stuart F. Schlossman .. et al., ed. Leucocyte typing V : white cell differentiation antigens : proceedings of the fifth international workshop and conference held in Boston, USA, 3-7 November, 1993. Oxford: Oxford University Press; 1995; . Clone B20.1 : Grégoire C, Rebaï N, Schweisguth F, et al. Engineered secreted T-cell receptor alpha beta heterodimers.. Proc Natl Acad Sci USA. 1991; 88(18):8077-81.

Pircher H, Rebaï N, Groettrup M, et al. Preferential positive selection of V alpha 2+ CD8+ T cells in mouse strains expressing both H-2k and T cell receptor V alpha a haplotypes: determination with a V alpha 2-specific monoclonal antibody.. Eur J Immunol. 1992; 22(2):399-404.

Tomonari K, Fairchild S, Rosenwasser OA. Influence of viral superantigens on V beta- and V alpha-specific positive and negative selection. Immunol Rev. 1993; 131:131-168.

Eukaryotic cell lines

Policy information about cell lines

Cell line source(s)

Jurkat, Clone E6-1 ATCC ® TIB-152™ transduced to express MART-1 TCR receptor and NY-ESO-1 TCR receptor by Mark Yarmarkovich. Murine B4.2.3 was obtained from Dr. Kanann Natarajan (NIAID, NIH).

Authentication

In house the Jurkat Mart-1 line was authenticated by human CDS and MART-1 TCR expression. Jurkat NY-ESO-1 line authenticated by CDS expression and NY-ESO-1 expression. The murine B4.2.3 was a gift from Dr. Kannan Natarajan (NIAID, NIH) and authenticated in-house by B4.3.2 receptor expression.

Mycoplasma contamination

Cell lines were tested negative for mycoplasma contamination using PCR (Universal Mycoplasma Detection kit ATCC) Cat 30-1012k

Commonly misidentified lines
(See ICLAC register)

no commonly misidentified cell lines were used in the study.

Flow Cytometry

Plots

Confirm that:

☒ The axis labels state the marker and fluorochrome used (e.g. CD4-FITC).

☒ The axis scales are clearly visible. Include numbers along axes only for bottom left plot of group (a 'group' is an analysis of identical markers).

☒ All plots are contour plots with outliers or pseudocolor plots.

☒ A numerical value for number of cells or percentage (with statistics) is provided.

Methodology

Sample preparation

Cell lines were cultured in IDMEM with 10% FBS, 2mM Glutamine, SO 1.U./ml penicillin and SO µg/ml streptomycin, at 37C S% Co2, SS% humidtity. All staining was performed on live unfixed cells at 4C in PBS, 1% BSA, 2 mM EDTA.

Instrument

Flow Cytometry was performed on an LSR II (Becton Dickinson). Cell sorting was performed on FACS Aria FUSION (Becton Dickinson Biosciences).

Software	Collection software: BD FACSDiva'" Software, Becton Dickinson, San Jose Ca. Analysis software: FlowJo BD Biosciences, CA.
Cell population abundance	Post sort abundance of Jurkat DMFS MART-1 was> 9S% as determined by anti-CDS and MART-1 tetramer co- staining.<br>Post sort abundance of Jurkat NY-ESO-1 was confirmed by anti-CDS and NY-ESO-1 tetra mer staining as> SO% after two rounds of sorting. B4.2.3 cells were confirmed to be greater than 9S% abundant by staining with anti-V2alphaTCR antibody.
Gating strategy	Initial FCS/SSC voltage settings were selected using unstained cells to focus on the most abundant live singelton lymphocyte population (of lymphocyte cell lines). For cell sorting experiments, doublets were excluded by side and forward scattering properties comparing SSC-A and SSC-W, and FSC-W- and FCS-A. For sorting and analysis, dead cells were excluded by propidium iodide uptake. For human lymphocyte lines, anti-CDS co-staining was used to confirm cell viability as well as block non-specific tetramer staining. Positive gates and boundaries were determined by comparison with un-stained and mismatched cell populations. For human lymphocytes (Jurkat lines), positive staining was defined by upper quartile co staining with anti-CDS and specific tetra mer positive. For the murine B4.2.3 line, gating was determined by a standard comparison of staining of TCR positive cells with matched and mismatched peptide loaded tetramers.

☒ Tick this box to confirm that a figure exemplifying the gating strategy is provided in the Supplementary Information.
